# When supervisor support is not enough: the roles of impostor syndrome and neuroticism in academic procrastination among doctoral students in China

**DOI:** 10.3389/fpsyt.2026.1881973

**Published:** 2026-08-20

**Authors:** Xiaohan Yang, Chong Zhang, Min Xu, Kee Jiar Yeo, Yu Liu

**Affiliations:** 1 Department of Psychology and Special Education, China National Academy of Educational Sciences, Beijing, China; 2 Department of Educational Studies and Behavioral Sciences, Faculty of Educational Sciences and Technology, Universiti Teknologi Malaysia, Johor Bahru, Malaysia; 3 School of Humanities, Southwest Jiaotong University, Chengdu, China

**Keywords:** academic procrastination, academic stress, doctoral education, higher education, impostor syndrome, mixed-methods, supervisor support

## Abstract

**Introduction:**

Academic procrastination is a persistent challenge in doctoral education, yet the role of supervisor support remains unclear. This study examined the associations among supervisor support, impostor syndrome, neuroticism, and academic procrastination among Chinese doctoral students.

**Methods:**

An explanatory sequential mixed-methods design was adopted. Survey data were collected from 962 doctoral students and analysed using partial least squares structural equation modelling (PLS-SEM), followed by interviews with 57 participants to further explain the quantitative findings.

**Results:**

Supervisor support was not directly associated with academic procrastination but showed a significant, modest indirect association through impostor syndrome (β = −0.063, p < 0.05). Impostor syndrome and neuroticism were both positively associated with procrastination, whereas neuroticism did not moderate the association between supervisor support and procrastination. Interview findings indicated that self-doubt, perfectionism, and overthinking could sustain procrastination even in supportive supervisory contexts, while recognition and clear feedback helped students interpret support more constructively.

**Discussion:**

The findings refine social support theory in doctoral education by suggesting that supervisor support is linked to procrastination primarily through students’ competence-related self-evaluations rather than through a direct behavioural pathway. In practice, supervisory support should be combined with interventions targeting impostor feelings and self-regulation.

## 1 Introduction

The mental health and academic functioning of doctoral students have become an increasingly prominent concern in higher education, particularly in systems undergoing rapid expansion. In China, doctoral admissions increased from 125,800 in 2021 to 171,100 in 2024, while the number of enrolled doctoral students rose from 509,500 to 676,300, reflecting a sustained expansion of doctoral training and a growing population facing prolonged academic and supervisory demands (1, 2). Recent evidence further shows that doctoral students’ well-being is shaped by multiple interacting factors across individual, interpersonal, academic, and organisational levels, and that psychological distress remains a persistent issue in doctoral education (3, 4). Against this backdrop, academic procrastination (AP) warrants closer attention because it not only delays task completion but may also undermine both academic functioning and psychological well-being.

Compared with undergraduates, doctoral students work in a less structured and more self-directed academic environment that requires sustained autonomy, research competence, and tolerance for uncertainty (5). They are expected to formulate research questions, manage long-term projects, publish high-quality work, participate in academic exchange, and make original scholarly contributions, often while navigating ambiguous timelines and strong evaluative pressure. Recent research indicates that the PhD student-supervisor relationship has substantial implications for students’ academic achievement, well-being, and professional development, making supervisor support a particularly salient contextual factor in doctoral study (6). In the Chinese context, emerging qualitative evidence further suggests that supervisory and group cultures may shape doctoral students’ stress, burnout, self-criticism, and emotional experiences in consequential ways (7).

Although these pressures are not unique to China, their scale and pace differ across doctoral systems. Internationally, research on doctoral education has documented persistent challenges - high attrition, prolonged time-to-completion, and elevated rates of psychological distress - that are closely tied to the quality of supervision and the demands of independent research. For instance, a large representative study of PhD students in Belgium found that roughly one in three was at risk of a common psychiatric disorder, with the supervisor’s leadership style among the organizational factors most strongly associated with mental health (8). At the same time, supervisory structures, funding arrangements, and cultural expectations vary substantially across countries, shaping how doctoral students perceive and benefit from supervisor support (5). Beyond procrastination, supervisor support has been linked to doctoral students’ broader academic outcomes through psychological mechanisms; for example, supportive supervision predicts research productivity via academic engagement and psychological capital among international doctoral students in China (9, 10).

In this study, academic procrastination is defined, following Steel (11), as the voluntary and unnecessary delay of an intended academic task despite anticipating negative consequences for doing so. In the doctoral context, this refers specifically to delaying the initiation or completion of research-related tasks—such as developing research questions, writing, conducting data analysis, and meeting programme milestones—even when students intend, and are expected, to proceed. Recent evidence suggests that procrastination is closely tied to fear of failure, perfectionism, emotional dysregulation, and other forms of psychological vulnerability, and that it is consistently associated with poorer academic outcomes and worse mental health (12). Although doctoral students in China have only recently begun to receive focused attention in procrastination research, existing evidence already points to the relevance of supervisory support for doctoral procrastination (13). However, important questions remain unresolved. First, it is unclear how procrastination operates in the distinctive, self-directed context of doctoral study as opposed to undergraduate settings. Second, it remains uncertain how doctoral procrastination is shaped by supervisor relationships and broader cultural-institutional conditions. Third, the psychological mechanisms explaining why some high-achieving doctoral students continue to delay essential academic tasks - despite adequate ability and support - are not yet well understood. Addressing these questions is especially important in rapidly expanding doctoral systems such as China.

### 1.1 Supervisor support and academic procrastination

Although academic procrastination has been widely studied, recent evidence suggests that its antecedents in doctoral education are more relational and context-dependent than those typically identified in undergraduate settings. In the Chinese doctoral context, supervisor support has been shown to be closely associated with academic procrastination, but its effects vary across support types. Specifically, academic support was directly and negatively associated with doctoral students’ procrastination, autonomy support was primarily associated with procrastination through indirect pathways, and emotional support showed no significant direct or indirect association with procrastination (13). These findings indicate that supervisor support is a multidimensional resource comprising components such as involvement, autonomy, and structure, which may operate through somewhat distinct mechanisms. Building on this work, the present study examines supervisor support as an overall relational resource and tests its direct and indirect associations with academic procrastination through impostor syndrome.

Recent studies further indicate that the supervisor-student relationship is one of the most consequential features of doctoral education. Qualitative evidence from Hong Kong shows that the PhD student-supervisor relationship affects students’ academic achievement, study progress, well-being, and career and social network development (6). In parallel, research on Chinese postgraduate students has found that perceived supervisor support is negatively associated with academic procrastination, with this relationship mediated by basic psychological needs satisfaction and learning engagement (14). Taken together, these findings suggest that supervisor support may reduce procrastination not simply by monitoring deadlines or providing technical advice, but by strengthening students’ sense of competence, motivational resources, and sustained engagement in academic work.

### 1.2 Mediating role of impostor syndrome

Impostor syndrome (IS), more precisely referred to in much of the recent literature as the impostor phenomenon, is another key psychological factor that may help explain academic procrastination (AP), especially in doctoral education. It was originally defined as a pattern in which high-achieving individuals doubt their accomplishments, struggle to internalize success, and fear being exposed as intellectual “frauds” despite objective evidence of competence (15). More recent reviews further indicate that impostor phenomenon is not an isolated feeling of insecurity but a broader pattern of persistent self-doubt that is closely linked to distress, maladaptive self-evaluation, and impaired functioning across academic and professional settings (16). In doctoral education specifically, impostor feelings appear to be widespread and strongly associated with poorer mental health, including higher levels of anxiety, depression, and loneliness (17).

Recent evidence suggests that impostor feelings are closely tied to perfectionistic concerns, fear of failure, and avoidant coping, making them highly relevant to academic procrastination (18). From this perspective, IS may function not merely as a co-occurring emotional difficulty, but as a psychological mechanism through which self-doubt is translated into task delay.

Supervisor and broader social support are therefore especially important in understanding IS. In doctoral education, recent evidence shows that greater supervisor empathy is significantly associated with lower impostor syndrome among doctoral students (19). Related research in higher education also suggests that supportive environments can mitigate impostor feelings indirectly by reducing anxiety and stress, highlighting the importance of emotionally validating and psychologically safe relationships (20). For doctoral students working in highly evaluative and uncertain academic settings, supportive supervision may thus help reduce procrastination not only by offering instrumental guidance, but also by weakening the impostor-related self-doubt that makes academic tasks feel threatening or overwhelming.

### 1.3 Moderating role of neuroticism

Neuroticism, a central dimension of the Big Five personality model, is generally characterized by heightened stress reactivity, frequent negative affect, rumination, and a tendency to respond strongly to adverse events (21, 22). These features make it especially relevant for understanding how students experience academic pressure and regulate their behaviour under demanding conditions (22).

In academic settings, neuroticism has been linked to academic procrastination and related emotion-regulation difficulties (23, 24). These findings suggest that neuroticism may matter not simply because it reflects general distress, but because it is tied to emotional instability, self-doubt, and avoidance tendencies that make sustained academic engagement more difficult.

Neuroticism may also shape how students perceive and use supportive resources. Research on graduate students in China has shown that higher neuroticism is associated with lower social support and less positive coping, and that these factors partly explain the link between neuroticism and poorer psychological outcomes (22). This suggests that highly neurotic students may be less likely to experience support as fully reassuring or actionable, especially under conditions of academic uncertainty and evaluation pressure (22). More broadly, recent work with medical postgraduates indicates that personality traits can moderate the relationship between academic stress and academic achievement, supporting the broader view that dispositional characteristics may condition how students respond to contextual demands and resources, even though evidence for neuroticism specifically remains mixed (25). On this basis, it is reasonable to hypothesise that neuroticism may moderate the effectiveness of supervisor support in reducing academic procrastination among doctoral students.

### 1.4 Theoretical framework

Social support theory posits that supportive interpersonal relationships promote functioning through both direct and buffering effects (26). Recent evidence in higher education is broadly consistent with this view, showing that social support can reduce impostor-related distress by alleviating anxiety and stress (20).

In doctoral education, supervisor support can be understood as a particularly salient form of social support because supervisory relationships are central to students’ academic progress and emotional experience. Rather than functioning only as a source of technical guidance, supervisor support may also shape how doctoral students interpret academic demands, evaluate their own competence, and cope with uncertainty (27). From this perspective, supervisor support is theoretically relevant to the present study because it may influence academic procrastination both directly, as a contextual resource, and indirectly, through students’ impostor feelings and self-regulatory difficulties.

**Figure 1:**
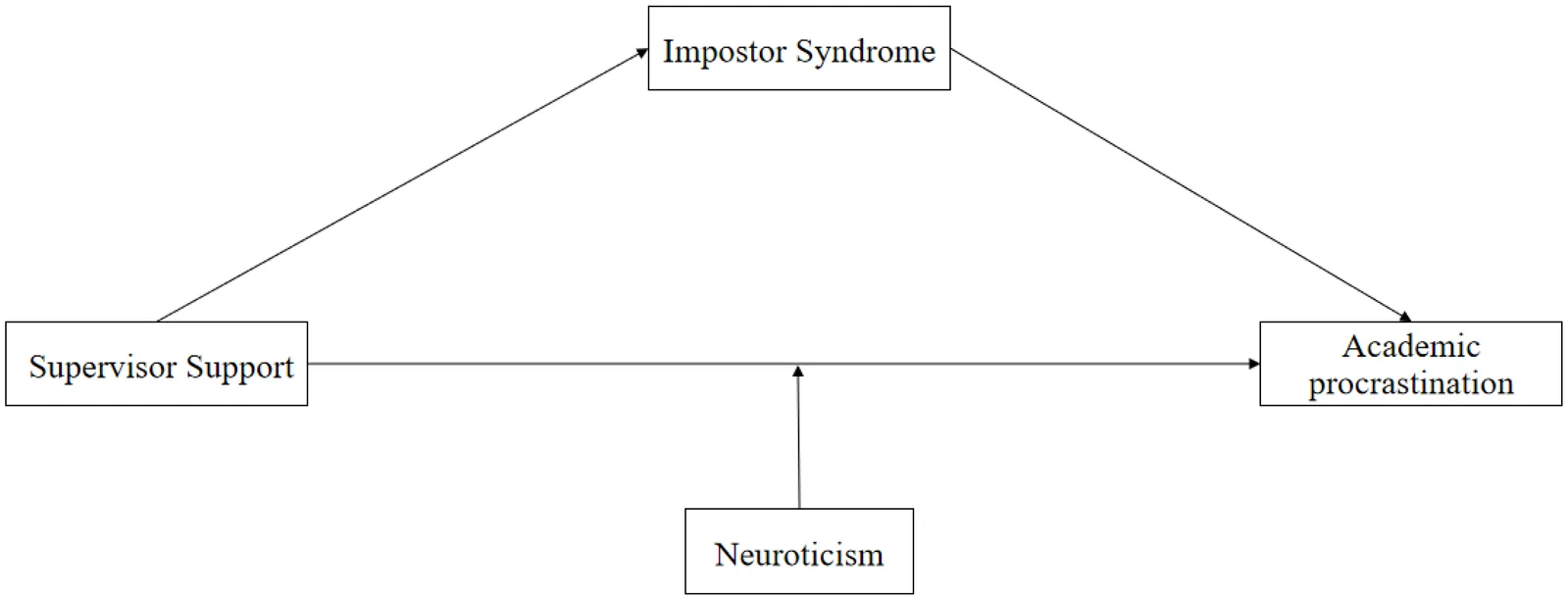
Conceptual Model for Quantitative Analysis.

At the same time, the effectiveness of support is unlikely to be uniform across individuals. Recent research on graduate students indicates that neuroticism is associated with lower perceived social support and less adaptive coping, and that social support partly mediates the relationship between neuroticism and depressive symptoms (22). This suggests that personality traits such as neuroticism may shape how doctoral students perceive, internalize, and benefit from supervisor support, particularly under conditions of uncertainty and evaluation pressure. On this basis, the present study proposes the conceptual framework shown in **Figure 1** to examine the direct and indirect associations among supervisor support, impostor syndrome, neuroticism, and academic procrastination. Specifically, we hypothesize that supervisor support is negatively associated with academic procrastination and impostor syndrome, that supervisor support has an indirect association with procrastination through impostor syndrome, and that neuroticism is positively associated with procrastination and moderates the supervisor support-procrastination association.

These assumptions lead to the following hypotheses:

H1: Supervisor support is negatively related to academic procrastination.
H2: Supervisor support is negatively related to impostor syndrome.
H3: Impostor syndrome is positively related to academic procrastination.
H4: Supervisor support is indirectly related to academic procrastination through impostor syndrome.
H5: Neuroticism is positively related to academic procrastination.
H6: Neuroticism moderates the association between supervisor support and academic procrastination.

## 2 Method

### 2.1 Participants and procedure

This study adopted a sequential explanatory mixed-methods design, in which a quantitative survey was followed by qualitative interviews to further interpret the statistical results (28). In the quantitative phase, data were collected from Chinese doctoral students using a combination of convenience and snowball sampling. To be eligible, participants had to be doctoral students currently enrolled at a Chinese university. The questionnaire was built on the Wen Juan Xing platform and distributed through doctoral programme coordinators and academic social media groups; coordinators forwarded the survey link to enrolled doctoral students, who could also pass it on to eligible peers. Recruitment targeted several universities located in major Chinese cities, including Beijing, Shanghai, Guangzhou, and Nanjing, and the survey was open to doctoral students across academic disciplines. A total of 962 valid responses were retained for analysis. Participation was voluntary; students read an information sheet, provided informed consent, and were assured of anonymity and confidentiality in line with ethical guidelines.

In the qualitative phase, 57 survey respondents who had indicated willingness to be interviewed were purposively selected to capture variation in gender, year of study, and levels of neuroticism, impostor syndrome, and academic procrastination. Semi-structured online interviews were conducted, audio-recorded with consent, transcribed verbatim and analysed to explore experiences of supervisor support, impostor feelings and procrastination, thereby complementing the quantitative findings (29). During the preparation of this manuscript, the authors used Claude Sonnet 4.5 solely to assist with English-language translation and stylistic editing. The authors reviewed and revised all AI-assisted output and take full responsibility for the content of the article. No generative AI tool was used to formulate the research questions, analyse the data, interpret the findings, or draw the conclusions.

### 2.2 Instrumentation for quantitative phase

In the quantitative phase, supervisor support was measured using the Need Support Scale (NSS) (30), which captures three dimensions of support from supervisors, namely involvement (INV), autonomy (AUTO), and structure (STRU). The NSS was selected because it was developed and validated specifically for the doctoral supervisory context rather than for general workplace support, and because it has been applied in comparable research on Chinese doctoral students (13); this made it more appropriate than more general measures such as the Eisenberger perceived support scale. A sample item is “My supervisor is concerned about me, not only as a researcher but also as an individual.” The original scale showed acceptable reliability (α = 0.62-0.85; test-retest = 0.64-0.68) (30), and in this study the Cronbach’s alpha for the total scale was 0.936. Consistent with our theoretical focus on supervisor support as an overall relational resource, and given the high internal consistency of the total scale, supervisor support was modeled as a higher-order construct comprising the three first-order dimensions.

Neuroticism was assessed with the Neuroticism subscale of the Big Five Inventory (BFI-N) (31), capturing doctoral students’ experiences of anxiety, self-doubt, mood swings, and stress. The BFI-N was selected because it is a widely used, cross-culturally validated measure of neuroticism with an established Chinese version, allowing comparability with prior research on graduate students. A sample item is “I see myself as someone who gets nervous easily.” The original inventory has demonstrated good internal consistency (α = 0.75-0.90) and high test-retest reliability (0.80-0.90) (31). In this study, the Chinese version of the BFI-N yielded a Cronbach’s alpha of 0.912.

Impostor syndrome was measured using the Short Clance Impostor Phenomenon Scale (CIPS-10) (32), a 10-item condensed version of the Clance IP Scale (33) adapted for doctoral students and rated on a 1 (Never) to 5 (Always) Likert scale. The CIPS-10 was chosen because it preserves the validated content of the original Clance scale while reducing respondent burden, and because the short form has been validated for doctoral student samples (32). A sample item is “I am afraid that people important to me may find out I am not as capable a doctoral student as they assume.” The original scale showed very high internal consistency (α = 0.93) (32), and the Cronbach’s alpha in this study was 0.919.

Academic procrastination was assessed with the behavioral subscale of the Behavioral and Emotional Academic Procrastination Scale (BEAPS) (34), comprising three items on delays in initiating research-related tasks. The BEAPS was selected because it was recently developed and validated specifically to measure academic procrastination, and its behavioral subscale directly targets task-initiation delay, which is central to the doctoral procrastination examined here. A sample item is “I unnecessarily waste a lot of time before I start working on my research-related tasks.” In the original validation, internal consistency for the factors ranged from 0.86 to 0.89, with test-retest reliability between 0.52 and 0.62 (34). In this study, Cronbach’s alpha was 0.889.

The quantitative data were analyzed using PLS-SEM in SmartPLS 4.0 to test the hypothesized direct, mediating, and moderating effects. The mediating role of impostor syndrome was examined with a bias-corrected bootstrap procedure (10,000 resamples) following Preacher and Hayes (35, 36). The moderating effect of neuroticism was assessed using an interaction term between neuroticism and supervisor support in SmartPLS (37). Analyses followed a two-step approach: the measurement model was first assessed for reliability and validity (a confirmatory step), after which the structural model was estimated to test the hypotheses. No exploratory factor analysis was conducted, as all constructs were measured with previously established and validated scales.

### 2.3 Instrumentation and procedure for qualitative phase

The qualitative phase used semi-structured interviews to further explain and contextualise the quantitative findings within the sequential explanatory design (28, 29). Interviews were designed to explore participants’ accounts that could contextualise the non-significant direct association between supervisor support and academic procrastination and the observed indirect association through impostor syndrome, using open-ended questions about experiences of supervisor support, impostor feelings, procrastination and emotional responses to academic stress, with follow-up probes for concrete examples. A purposive sample of 57 survey respondents was selected to capture diversity in neuroticism, impostor syndrome, procrastination, demographic backgrounds and supervisory experiences. Each interview, conducted in Chinese, lasted approximately 60–90 minutes, was audio-recorded with consent and transcribed verbatim.

Data were analysed using Braun and Clarke’s (38) thematic analysis framework in NVivo 15.0. Initial open coding identified key concepts and patterns, followed by focused coding to develop broader themes. Two researchers independently coded 20% of the transcripts and discussed discrepancies until consensus. Intercoder agreement, calculated as percentage agreement, was 90%, indicating satisfactory reliability. Trustworthiness was enhanced through member checking of transcripts and preliminary interpretations, peer debriefing with colleagues not involved in the fieldwork, and detailed documentation of analytic decisions (38). Integrating the qualitative themes with the quantitative results strengthened the validity of the conclusions (28) and allowed for culturally sensitive interpretation in the context of Chinese doctoral education.

## 3 Quantitative results

### 3.1 Data screening and descriptive analysis

After removing 10 straight-line responses and 2 outliers, 962 valid questionnaires were retained for analysis. Overall, 66.11% of participants were female (n = 636), 33.68% were male (n = 324), and 0.21% identified as non-binary (n = 2). Most participants were aged 26-30 (46.82%, n = 451) or 31-40 (38.05%, n = 366), while 12.68% (n = 122) were aged 25 or younger and 2.39% (n = 23) were aged 41 or older.

According to the CIPS-10, 37.55% of doctoral students reported moderate IS (n = 361), 31.23% frequently experienced IS (n = 300), and 11.39% reported intense IS (n = 110), while only 19.83% (n = 191) reported little or no IS. Among participants reporting frequent and intense IS, women accounted for 72.33% (n = 217) and 78.18% (n = 86), respectively, a higher proportion than their 66.11% share of the overall sample.

### 3.2 Common method variance

In this study, common method variance (CMV) was a potential concern because all independent and dependent variables were self-reported at the same time (39). We addressed this using a single common method factor based on the PLS marker-variable technique. Seven items collected in the same survey but not included in the substantive model were selected as marker indicators and specified as exogenous predictors of each endogenous variable to form a latent method factor. Comparison of the method-factor model with the baseline model showed that all previously significant paths remained significant, indicating that CMV was unlikely to bias the results.

### 3.3 Data analysis

The minimum sample size for the study was determined using G*Power software (40). An *a priori* power analysis was conducted in G*Power for a fixed-effects multiple regression model with four predictors. Assuming a medium effect size (f² = 0.15), an α level of 0.05, and statistical power of 0.80, the minimum required sample size was 85. The final sample of 962 participants therefore exceeded the minimum requirement. Therefore, the sample size of 962 Chinese doctoral students was sufficient for data analysis.

#### 3.3.1 Measurement model

Following Anderson and Gerbing’s (41) recommendation, the model was tested using a two-step approach. First, the measurement model was tested to assess the validity and reliability of the instruments used, as per the guidelines by Hair et al. (37), and then the structural model was run to test the developed hypotheses.

For the measurement model, loadings, Average Variance Extracted (AVE), and Composite Reliability (CR) were evaluated. Loadings should be ≥0.5, AVE should be ≥0.5, and CR should be ≥0.7. As shown in **Table 1**, all AVEs were above 0.5 and CRs were above 0.7. The loadings were also acceptable, with only one loading less than 0.708 (37).

**Table 1:** Measurement model.

First order constructs	Dimension	Items	Loadings	AVE	CR
Supervisor Support	Involvement	INV1	0.941	0.875	0.965
		INV2	0.918		
		INV3	0.943		
		INV4	0.939		
	Autonomy	AUTO1	0.756	0.601	0.819
		AUTO2	0.749		
		AUTO3	0.820		
	Structure	STR1	0.925	0.876	0.966
		STR2	0.941		
		STR3	0.939		
		STR4	0.938		
Neuroticism	—	NEU1	0.759	0.621	0.929
		NEU2	0.780		
		NEU3	0.822		
		NEU4	0.711		
		NEU5	0.863		
		NEU6	0.716		
		NEU7	0.792		
		NEU8	0.845		
Impostor Syndrome	—	IS1	0.733	0.580	0.932
		IS2	0.784		
		IS3	0.770		
		IS4	0.808		
		IS5	0.809		
		IS6	0.818		
		IS7	0.742		
		IS8	0.818		
		IS9	0.705		
		IS10	0.732		
Academic Procrastination	—	AP1	0.887	0.849	0.931
		AP2	0.932		
		AP3	0.896		

AVE, Average Variance Extracted; CR, Composite Reliability. Supervisor Support was modeled as a higher-order construct comprising three first-order dimensions: involvement, autonomy, and structure.

Supervisor support was specified as a higher-order construct using the two-stage approach (37), with involvement, autonomy, and structure as its lower-order dimensions; all three met the reliability and validity criteria reported in **Table 1**.

In the second step, discriminant validity was assessed using the Heterotrait-Monotrait (HTMT) criterion proposed by Henseler et al. (42) and updated by Franke and Sarstedt (43). The stricter criterion for HTMT values is ≤ 0.85, while the more lenient criterion is ≤ 0.90. As indicated in **Table 2**, all HTMT values were below the stricter threshold, suggesting that the respondents perceived the constructs as distinct from each other. Although the HTMT value between neuroticism and impostor syndrome was somewhat higher (0.747), it remained below the conservative 0.85 threshold, indicating that the two constructs were empirically distinguishable in this sample while still being moderately related, consistent with the theoretical view that neurotic dispositions may predispose students to impostor feelings. Overall, these tests of validity indicate that the measurement items are both valid and reliable.

**Table 2:** Discriminant validity (HTMT).

Constructs	1	2	3	4
1. Supervisory Support				
2. Neuroticism	0.181			
3. Impostor Syndrome	0.155	0.747		
4. Academic Procrastination	0.172	0.567	0.653	

#### 3.3.2 Structural Model

Based on the recommendations of Hair et al. (37) and Cain et al. (44), we assessed multivariate skewness and kurtosis. Mardia’s multivariate skewness (β = 1.279, p < 0.01) and kurtosis (β = 25.990, p < 0.01) indicated that the data were not multivariate normal. Following Becker et al. (45), we therefore used a 10,000-sample bootstrapping procedure to obtain path coefficients, standard errors, t-values and p-values for the structural model. In line with Hahn and Ang’s (46) critique that p-values alone are not a robust criterion, we interpreted results using a combination of p-values, confidence intervals and effect sizes (see **Table 3** for the standards used to test the hypotheses).

**Table 3:** Hypothesis testing direct effects.

Hyp.	Rel.	β	SE	t	5%	95%	f2
H1	SS→AP	-0.053	0.060	0.896	-0.147	0.050	0.004
H2	SS→IS	-0.140*	0.073	1.932	-0.261	-0.022	0.020
H3	IS→AP	0.449***	0.080	5.591	0.316	0.578	0.176
H5	NEU→AP	0.199**	0.072	2.763	0.084	0.322	0.034
H6	NEU×SS→AP	0.063	0.058	1.094	-0.034	0.156	0.007

Bootstrapping was conducted with 10,000 resamples. LL, lower limit; UL, upper limit. *p < 0.05, **p < 0.01, ***p < 0.001 (one-tailed).

Consistent with the research hypotheses, we examined the relations among the predictive factors. Supervisor support (SS) was not significantly associated with academic procrastination (AP; β = -0.053, p > 0.05), so H1 was not supported. SS was negatively associated with impostor syndrome (IS; β = -0.140, p < 0.05), supporting H2, whereas IS was positively associated with AP (β = 0.449, p < 0.001), supporting H3. Neuroticism was also positively associated with AP (β = 0.199, p < 0.01), supporting H5. However, the interaction between neuroticism and SS was not significant (β = 0.063, p > 0.05), so H6 was not supported.

To test the mediation hypothesis, bootstrapping for indirect effects was conducted following the recommendations of Preacher and Hayes (35, 36). A significant mediating effect can be concluded if the confidence interval does not cross zero. As shown in **Table 4**, the indirect association between SS and AP through IS was negative and statistically significant (β = -0.063, p < 0.05), supporting H4. Given the cross-sectional design, this result is interpreted as a statistical indirect association rather than evidence of a causal or temporally ordered mediation process.

**Table 4:** Hypothesis testing indirect effects.

Hyp.	Rel.	β	SE	t	5%	95%
H4	SS→IS→AP	-0.063*	0.036	1.731	-0.128	-0.009

Bootstrapping was conducted with 10,000 resamples. LL, lower limit; UL, upper limit. *p < 0.05, **p < 0.01, ***p < 0.001 (one-tailed).

## 4 Qualitative results

To deepen understanding of the quantitative results, we conducted semi-structured interviews with doctoral students. After the survey, 57 respondents were purposively selected based on their survey responses to capture variation in impostor syndrome, neuroticism, and academic procrastination and to discuss their experiences of supervisor support, impostor feelings, and task delay. Fifty-seven students agreed to take part in online interviews after being informed about the study and confidentiality.

The interview protocol was informed by the survey findings and focused on how students experienced and managed impostor feelings, interacted with supervisors and dealt with procrastination. All interviews were conducted online, audio-recorded with consent and transcribed verbatim. The data were analysed thematically using NVivo 15, yielding three main themes (see **Table 5**).

**Table 5:** Main themes and subthemes from qualitative analysis.

	Main themes	Subthemes
1	Supervisor Support Dynamics	Supportive practices (clear guidance, regular feedback, structured deadlines)
		Detrimental practices (dismissive interactions, vague feedback, overly critical responses)
		Impact on task motivation and completion
		Balance between support and independence
2	Manifestations of Impostor Syndrome	Response to constructive support (validation, confidence building)
		Impact of critical feedback
		Reactions to excessive supervision
		Relationship between recognition and self-doubt
3	Neuroticism’s Influence on Academic Experience	Anxiety-driven procrastination
		Perfectionism and task avoidance
		Response to structured guidance
		Persistence of self-doubt despite support

### 4.1 Supervisor support in relation to academic procrastination and impostor syndrome

#### 4.1.1 Perceived supervisor support and academic task completion

Supervisor support plays an important role in shaping doctoral students’ academic behaviors, particularly academic procrastination. Positive supervisory relationships were often described as sources of motivation and accountability, whereas perceived neglect or criticism were associated with uncertainty and delays in task initiation.

Several participants emphasized the motivational role of clear guidance and regular feedback. One participant shared:

“My supervisor always sets clear deadlines and gives helpful feedback whenever we meet. It keeps me on track because I know exactly what they expect from me.”

Such structured practices help reduce task ambiguity and make it easier for students to stay engaged with their work.

However, many participants also reported dismissive or critical interactions that left them feeling unsupported. One doctoral student reflected:

“Whenever I approach my supervisor with a problem, they say, ‘How can you not know this? You’re a PhD student!’ It makes me feel lost and hesitant to ask for help again.”

Another participant expressed frustration with a lack of actionable advice:

“When the feedback is vague, it feels like I’m left to figure everything out on my own. I start second-guessing myself and end up putting things off because I’m not sure if I’m heading in the right direction.”

At the same time, participants stressed that procrastination often stemmed from their own habits and thought patterns rather than external factors such as supervisor support. One participant noted:

“No matter how much my supervisor pushes me or sets deadlines, I still find ways to delay things until the very last moment. It’s not about them-it’s my own struggle to stay focused I think.”

Another participant shared a similar perspective:

“Even if I know what’s expected, I sometimes can’t help but procrastinate. I’ll find excuses or other tasks to justify putting things off. It’s not something my supervisor can fix for me, but I need to work on myself.”

The interviews suggest that supervisor support is a double-edged resource: clear and constructive guidance can motivate academic task completion, whereas procrastination often remains a self-driven self-regulation problem rooted in internal cognitive and emotional patterns (11, 47, 48). This pattern is consistent with the quantitative finding that supervisor support was not directly associated with procrastination (H1 not supported). Participants frequently experienced procrastination as being connected to internal cognitive and self-regulatory difficulties, suggesting that the availability of supervisor support alone was not always accompanied by timely task initiation.

#### 4.1.2 Perceived supervisor support and impostor feelings

Supervisor support emerged as a key influence on IS among doctoral students: constructive interactions tended to ease self-doubt, whereas critical or overly intense supervision often intensified it.

Participants frequently described how supportive supervisors reduced impostor feelings. One participant remarked:

“ When I feel unsure, their reminders about my progress and achievements make a big difference—it helps me believe I belong here.”

Conversely, participants noted that overly critical supervisors or a lack of acknowledgment heightened their IS. One participant explained:

“When my supervisor only points out flaws and doesn’t acknowledge what I’ve done right, it’s disheartening. It makes me question whether I’m capable and whether I should even be in the program.”

Negative feedback that is not balanced with positive reinforcement can undermine students’ self-perception and exacerbate impostor-like feelings, leading them to interpret difficulties as personal failure rather than situational challenge.

Some participants also reported that excessive attention from supervisors could amplify IS. One participant shared:

“Sometimes, my supervisor’s frequent check-ins and high expectations feel overwhelming. Instead of feeling supported, I start doubting whether I can meet their standards, and that stress makes it hard to focus.”

Thus, even well-intentioned engagement may heighten pressure and self-doubt if experienced as over-monitoring. Supervisor support has the potential to reduce IS when it combines recognition of accomplishments with constructive feedback, but dismissive or overly critical supervision, as well as excessive monitoring, may instead intensify self-doubt. These findings emphasize the need for tailored supervisory approaches that account for individual student needs (49). In practice, this requires distinguishing supportive engagement from excessive monitoring: supervisors may be most helpful when they balance involvement with respect for students’ autonomy, pairing recognition and constructive guidance with sufficient space for independent work, and calibrating the frequency and intensity of check-ins to each student’s needs rather than applying uniform oversight.

### 4.2 Emotional responses to supervisor support among doctoral students with neuroticism

#### 4.2.1 Neuroticism and academic procrastination

Participants with higher levels of neuroticism consistently reported a greater tendency to procrastinate, often due to heightened anxiety and fear of failure. One participant shared:

“Before starting any task, I think about all the ways it could go wrong. The more I overthink, the more I feel paralyzed, and I end up putting it off.”

This reflects that neurotic individuals are more likely to procrastinate due to their tendency to ruminate on potential negative outcomes. Their heightened emotional responses to perceived challenges exacerbate avoidance behaviors, leading to significant delays in task initiation. Another participant explained:

“If I’m not completely confident in what I’m doing, I can’t start. I keep waiting for the ‘perfect’ moment, but it rarely comes.”

The pursuit of perfection, combined with fear of mistakes, can turn procrastination into a short-term coping strategy with long-term costs. Steel and Klingsieck (48) note that this perfectionistic mindset, common among neurotic individuals, fosters decision paralysis and avoidance that undermine academic performance. Although structured guidance and empathetic feedback from supervisors or institutions may buffer these effects (50), our data suggest that entrenched cognitive and emotional barriers often limit what external support can achieve, highlighting the need for targeted interventions (e.g., cognitive-behavioral or stress-management training) to help neurotic students develop healthier coping strategies.

#### 4.2.2 Neuroticism in the context of supervisor support and academic procrastination

The accounts in this section came primarily from interviewees who had reported relatively high neuroticism in the survey, which allowed comparison of how more and less neurotic students experienced supervisor support. Although supervisor support was generally viewed as helpful, students with high neuroticism described mixed outcomes. For some, structured and empathetic supervision alleviated stress and reduced procrastination. One participant remarked:

“When my supervisor breaks tasks into smaller steps, it helps me feel less anxious and makes it easier to get started. That kind of structure really works for me.”

This illustrates how clear guidance and reduced ambiguity can ease the cognitive overload often experienced by highly neurotic individuals.

However, others felt that their neurotic tendencies overshadowed the benefits of supervisor support. One participant explained:

“Even when my supervisor gives me clear instructions, I overthink every detail. I’m constantly doubting myself, and their support doesn’t fully ease that.”

This highlights a broader challenge in supporting neurotic students: heightened self-doubt and rumination can prevent them from internalizing or acting on even constructive feedback. Neuroticism amplifies emotional distress and self-critical thinking, reducing the effectiveness of external interventions such as supervisor support. These mixed accounts are consistent with the absence of a statistically significant moderation effect (H6), because they did not indicate a uniform pattern in how students with higher neuroticism responded to supervisor support; while supportive supervisors can remove some barriers to task completion, deeply internalized doubt and overthinking often require additional targeted interventions, such as cognitive-behavioral or stress management programs, to foster healthier coping strategies.

### 4.3 Integration of quantitative and qualitative findings

Integration of the quantitative and qualitative findings indicated both convergence and extension. The interview accounts helped contextualise the non-significant direct association between supervisor support and procrastination, as participants attributed their continued task delay to self-doubt, perfectionism, and difficulties in self-regulation even when support was available. They also supported the negative association between supervisor support and impostor syndrome, as recognition and constructive feedback were commonly linked to greater confidence. Beyond the quantitative results, however, the interviews showed that support was not uniformly beneficial: excessive monitoring and predominantly critical feedback could intensify pressure and self-doubt. Similarly, the mixed responses of highly neurotic students to structured guidance were consistent with the absence of a stable moderating effect. Overall, the qualitative findings suggest that the meaning and perceived quality of supervisor support may be as important as its availability.

## 5 Discussion

This sequential explanatory mixed-methods study identified a differentiated pattern of associations with academic procrastination among Chinese doctoral students. Supervisor support was not directly associated with procrastination but was negatively associated with impostor syndrome, which was positively associated with procrastination. Neuroticism was also positively associated with procrastination but did not moderate the supervisor support-procrastination association. The qualitative findings help explain these results by showing that many students delayed academic tasks not because support was entirely absent, but because perfectionism, self-doubt, emotional avoidance, and persistent overthinking interfered with their ability to translate support into action.

The non-significant direct effect of supervisor support suggests that, in doctoral education, support may function less as an immediate behavioural control and more as a relational condition that shapes motivation, academic belonging, and the usability of guidance (26, 27). This interpretation is consistent with recent doctoral research showing that trust in one’s advisor prospectively predicts motivation, well-being, self-efficacy, academic belonging, and academic self-control over time (27). It also aligns with newer work on doctoral supervision indicating that supervisory interactions are deeply implicated in doctoral students’ academic socialisation, while the tone, clarity, and specificity of supervisory feedback influence how students interpret and act on guidance (51, 52). Read alongside the interview data in the present study, these findings suggest that support matters, but its effect on procrastination is indirect: support first has to be cognitively and emotionally converted into a sense of confidence, clarity, and controllability before it can influence action.

The mediating role of impostor syndrome is therefore one of the most theoretically important findings of this study. Rather than reducing procrastination outright, supervisor support appears to matter when it changes how students understand their competence, legitimacy, and right to belong in doctoral study. Recent doctoral research shows that IS is widespread and strongly associated with anxiety, depression, and loneliness, while self-compassion substantially weakens the link between impostor feelings and mental distress (17). This resonates closely with the qualitative evidence in the present study: students described that specific recognition, credible affirmation, and clear expectations helped them feel more legitimate and more able to begin difficult tasks, whereas vague criticism or purely deficit-focused feedback intensified self-doubt and hesitation. These patterns suggest that impostor syndrome is not merely a co-occurring emotional problem; it is a plausible self-regulatory mechanism through which relational experiences in supervision become translated into avoidance and task delay (17).

At the same time, the magnitude of this indirect effect was modest (β = -0.063), which carries its own practical message. Although statistically significant, the modest indirect association suggests that supervisor support alone is likely to have limited practical relevance for reducing procrastination. This is largely because the path from supervisor support to impostor syndrome was itself relatively weak (β = -0.140), even though impostor syndrome strongly predicted procrastination (β = 0.449). Notably, although small, this indirect effect (β = -0.063) was comparable in magnitude to, and slightly larger than, the non-significant direct effect of supervisor support on procrastination (β = -0.053). The small effect sizes for the supervisor-support paths reported in **Table 3** (Cohen’s f² = 0.004 for the direct path and 0.020 for the support-to-impostor path) are consistent with this pattern. Together, these results suggest that the limited association between supervisor support and procrastination was observed primarily through impostor syndrome rather than through a direct association. In practical terms, improving supervisory relationships may help ease impostor feelings and, indirectly, procrastination, but any practical benefit is likely to be incremental; supportive supervision alone is unlikely to resolve deeply internalized self-doubt, consistent with the interview accounts in which procrastination persisted despite supportive supervisors. Supervisor support should therefore be treated as one meaningful but partial lever, best combined with interventions that target impostor feelings and self-regulation more directly.

The Chinese doctoral context may help to interpret why supervisor support was associated with impostor syndrome but was not directly associated with procrastination. Doctoral supervision in China is often embedded in supervisor-centred and hierarchical relationships in which supervisors may control access to projects, academic resources, evaluation, and degree progression (52–54). Under such conditions, students may hesitate to question ambiguous feedback or disclose difficulties because doing so can be experienced as challenging authority or risking loss of face. Consequently, support may be valued at the relational level without necessarily being translated into help-seeking or timely task initiation. The interview accounts of vague criticism, intensive check-ins, and persistent self-doubt are consistent with this interpretation and with evidence that supervisor-group cultures are closely related to Chinese doctoral students’ stress and self-criticism (7). From a social support perspective, this suggests that the meaning and usability of support - not merely its availability - depend on the cultural and relational context in which it is delivered. However, these cultural processes were not directly measured, and Chinese doctoral education is not homogeneous; the proposed interpretation should therefore be tested across institutions, disciplines, and supervisory arrangements.

Neuroticism added a further psychological layer. Although higher neuroticism was associated with greater academic procrastination, it did not change the relationship between supervisor support and procrastination. This pattern suggests that neuroticism may function less as a boundary condition on the effectiveness of support and more as a relatively stable vulnerability factor that directly increases avoidance. One plausible explanation is that neuroticism heightens threat sensitivity, rumination, and negative affect, making academic tasks feel more burdensome even when support is available. From this perspective, the non-significant moderating effect does not mean that supervisor support is unimportant for highly neurotic students; rather, it suggests that the impact of support may be constrained by internally driven emotional processes. It is also possible that neuroticism influences procrastination partly through stronger impostor-related self-doubt, rather than by altering how supervisor support operates. This interpretation is *post-hoc*, however: the present model specified impostor syndrome as a mediator of supervisor support rather than of neuroticism, and the moderate association between neuroticism and impostor syndrome observed here (HTMT = 0.747) is consistent with, but does not establish, such a pathway. Formally testing a neuroticism-to-impostor-syndrome-to-procrastination model is therefore an important direction for future research.

Theoretically, this study makes three contributions. First, it refines the application of social support theory to doctoral education by showing that supervisor support was related to procrastination primarily through an indirect association with students’ self-evaluations rather than through a direct behavioural association. Second, it identifies impostor syndrome as a plausible self-regulatory pathway linking supervisory experiences with avoidance and task delay, extending procrastination research beyond its traditional emphasis on time management and trait self-control toward identity- and competence-related processes. Because the data were cross-sectional, this pathway should be understood as a theoretically informed interpretation rather than an established causal mechanism. Third, the findings distinguish supervisor support as a potentially malleable contextual resource from neuroticism as a relatively stable dispositional vulnerability, suggesting that the two are associated with procrastination in different ways.

Practically, the findings suggest that doctoral support should move beyond generic calls for “more support” and attend to the specific qualities of supervision that shape students’ self-evaluation. Because support appears to influence procrastination mainly by easing impostor feelings, supervisors may be most effective when they pair constructive, specific feedback with credible recognition of progress and clear, attainable expectations, rather than relying on monitoring or deadline pressure alone. Given that the indirect pathway was modest and that interview participants continued to procrastinate despite supportive supervision, supervisory practice should be complemented by interventions that target self-doubt and self-regulation more directly, such as self-compassion training, cognitive-behavioural strategies, and structured writing or milestone support. At the institutional level, this implies embedding accessible psychological support alongside supervisory training, so that students experiencing strong impostor feelings, perfectionism, or neurotic vulnerability are not left to rely on the supervisory relationship alone.

## 6 Conclusion

The findings indicate that academic procrastination among Chinese doctoral students cannot be understood solely in relation to the availability of supervisor support. Supervisor support was not directly associated with lower academic procrastination but showed a significant, modest indirect association through impostor syndrome. Neuroticism was positively associated with procrastination but did not moderate the supervisor support-procrastination association. These cross-sectional associations are consistent with the possibility that students’ self-evaluations and emotional vulnerabilities are relevant to how supervisory experiences relate to procrastination, but they do not establish a causal sequence. These findings suggest that supervisory support matters not primarily because it immediately changes behavior, but because it helps shape how doctoral students perceive their competence, legitimacy, and ability to cope with academic demands.

Several limitations should be noted. First, the quantitative data were cross-sectional, so the hypothesized directional and mediating relationships cannot be interpreted as causal; the proposed pathway from supervisor support through impostor syndrome to procrastination requires confirmation with longitudinal or experimental designs that can establish temporal order. Second, all constructs were measured through self-report, which may introduce common method and social desirability biases; although a marker-variable analysis suggested that common method variance was unlikely to bias the results, self-reported procrastination and impostor feelings remain susceptible to recall and self-presentation effects. Third, the sample was drawn through convenience and snowball sampling from doctoral students at several universities in major Chinese cities, so the findings may not generalize to doctoral students in other regions, institution types, or academic systems and supervisory structures. Fourth, the demographic information collected was limited to age, current PhD semester, and gender; other potentially relevant characteristics, such as institution type, academic discipline, and marital status, were not recorded and could not be examined. Fifth, supervisor support was modeled as an overall higher-order construct; although this was consistent with our theoretical focus, it did not capture how specific dimensions of support, namely involvement, autonomy, and structure, might relate differently to procrastination. Sixth, academic procrastination was assessed with the three-item behavioural subscale of the BEAPS; although this subscale directly targets task-initiation delay, its brevity may limit the content coverage of a multifaceted construct, and future studies could employ more comprehensive procrastination measures. Finally, although the qualitative data enriched the interpretation of the statistical results, they were intended to deepen explanation rather than to provide broad representativeness.

Overall, the findings suggest that efforts to reduce doctoral students’ academic procrastination should combine effective supervisory practices with more direct attention to students’ psychological experiences. In practical terms, doctoral support should place greater emphasis on clear and constructive feedback, psychologically safe supervisory relationships, and timely recognition of students’ progress, while also addressing impostor feelings, perfectionism, and emotion-related self-regulation difficulties.

Building on these limitations, future research could pursue several directions. Longitudinal and experimental designs would help clarify the causal and temporal structure of the relationships observed here, particularly the mediating role of impostor syndrome. Future studies could also disaggregate supervisor support into its component dimensions to examine whether involvement, autonomy, and structure operate through distinct pathways, and could incorporate a broader set of participant characteristics, including institution type, discipline, and marital status, to test the robustness and boundary conditions of the present findings. Cross-cultural comparisons would further clarify which patterns are specific to the Chinese doctoral context and which are more general. Finally, because impostor feelings and neuroticism are closely tied to anxiety and stress, future work could complement self-report measures with physiological stress markers such as cortisol, sleep, and heart rate variability; as all constructs here were self-reported, such objective indicators would help corroborate self-reported distress, address the common-method concerns noted above, and test whether the psychological pathways identified in this study have measurable physiological correlates. Future work could also evaluate interventions that integrate supervisory practice with psychological support, such as self-compassion or cognitive-behavioural programmes, to promote doctoral students’ academic progress and well-being.

## Data Availability

The raw data supporting the conclusions of this article will be made available by the authors, without undue reservation.

## References

[1] Ministry of Education of the People’s Republic of China . 2021年全国教育事业发展统计公报 [Statistical Bulletin on the Development of National Education in 2021] (2022). Available online at: https://www.cee.edu.cn/n171/n459/n472/c425314/content.html (Accessed June 15, 2026).

[2] Ministry of Education of the People’s Republic of China . 2024年全国教育事业发展统计公报 [Statistical Bulletin on the Development of National Education in 2024] (2025). Available online at: https://www.cee.edu.cn/n171/n459/n472/c804898/content.html (Accessed June 15, 2026).

[3] Guo Y Liu X . Mental health and well-being of doctoral students: a systematic review. Int J Ment Health Promotion. (2026) 28:1. doi: 10.32604/ijmhp.2025.074063

[4] Mahsood N Mahboob U Aleem S Bhatti A Durrani S Javed MA . Navigating the doctoral journey: a qualitative study on PhD scholars’ well-being in Pakistan. Pakistan J Med Sci. (2025) 41:1402–9. doi: 10.12669/pjms.41.5.11982 40469127 PMC12130926

[5] Sverdlik A Hall NC McAlpine L Hubbard K . The PhD experience: a review of the factors influencing doctoral students’ completion, achievement, and well-being. Int J Doctoral Stud. (2018) 13:361–88. doi: 10.28945/411342520217

[6] Li Y Xu W Chen J . PhD student-supervisor relationship and its impacts: a perspective of the interpersonal relationship model. Front Educ. (2025) 10:1570137. doi: 10.3389/feduc.2025.1570137

[7] Xie S Yu S Xu L . Supervisor-group culture, age and the stress-burnout mechanism: a qualitative study of Chinese doctoral students. Front Psychol. (2026) 17:1794711. doi: 10.3389/fpsyg.2026.1794711 41853821 PMC12992317

[8] Levecque K Anseel F De Beuckelaer A Van der Heyden J Gisle L . Work organization and mental health problems in PhD students. Res Policy. (2017) 46:868–79. doi: 10.1016/j.respol.2017.02.00838826717

[9] Khuram W Wang Y Ali M Khalid A Han H . Impact of supportive supervisor on doctoral students’ research productivity: the mediating roles of academic engagement and academic psychological capital. SAGE Open. (2023) 13:1–15. doi: 10.1177/21582440231185554

[10] Khuram W Wang Y Khan S Khalid A . Academic attitude and subjective norms effects on international doctoral students’ academic performance self-perceptions. J Psychol Afr. (2021) 31:145–52. doi: 10.1080/14330237.2021.190318837339054

[11] Steel P . The nature of procrastination: a meta-analytic and theoretical review of quintessential self-regulatory failure. psychol Bull. (2007) 133:65–94. doi: 10.1037/0033-2909.133.1.65 17201571

[12] Ramadhani E Setiyosari P Indreswari H Setiyowati AJ Putri RD . Academic procrastination: a systematic review of causal factors and interventions. J Behav Cogn Ther. (2026) 36:100552. doi: 10.1016/j.jbct.2025.10055238826717

[13] Meng F Zhao Y Zang Z . Academic procrastination among doctoral students in China: the role of supervisory support, research self-efficacy, and persistence intention. SAGE Open. (2025) 15:21582440251330221. doi: 10.1177/21582440251330221

[14] Wang L Wang G . Perceived supervisor support and academic procrastination in postgraduate students: roles of basic psychological needs satisfaction and learning engagement. Behav Sci. (2024) 14:1005. doi: 10.3390/bs14111005 39594305 PMC11591262

[15] Clance PR Imes SA . The imposter phenomenon in high achieving women: dynamics and therapeutic intervention. Psychotherapy: Theory Res Pract. (1978) 15:241–7. doi: 10.1037/h008600627371692

[16] Gisselbaek M Seyour M Saxena S Berger-Estilita J LaDonna KA Elia N . Rethinking the impostor phenomenon: an umbrella review of concept, context and interventions. Med Educ. (2025) 60(6):607–24. doi: 10.1111/medu.70076 41151996 PMC13129615

[17] Clarke BJ Hartley MT . Exploring relationships between self-compassion, impostor phenomenon, and mental health among doctoral students. Front Psychol. (2025) 16:1669075. doi: 10.3389/fpsyg.2025.1669075 41377086 PMC12685627

[18] Hill AP Gotwals JK . A meta-analysis of multidimensional perfectionism and impostor phenomenon. J Res Pers. (2025) 118:104639. doi: 10.1016/j.jrp.2025.10463938826717

[19] Slimi O Muscella A Marsigliante S Bahloul M . Correlation between impostor syndrome among doctoral students and supervisor empathy in Tunisia. Front Psychol. (2024) 15:1382969. doi: 10.3389/fpsyg.2024.1382969 39135869 PMC11317466

[20] Fatima M Qamar M Irfan H Malik H Batool F Shahzad J . Examining the role of social support in imposter phenomenon among medical students through mediation and moderation analysis of anxiety and stress. Discover Ment Health. (2025) 5:207. doi: 10.1007/s44192-025-00347-7 41310244 PMC12748390

[21] Wright AJ Haehner P Hopwood CJ Bleidorn W . A systematic review and taxonomy of neuroticism interventions for the general public. Pers Sci. (2026) 7:27000710261421531. doi: 10.1177/27000710261421531

[22] Wan P Hu J Yang Q . The chain mediating role of social support and positive coping between neuroticism and depressive symptoms among graduate students. Front Psychiatry. (2024) 15:1424983. doi: 10.3389/fpsyt.2024.1424983 39391090 PMC11465235

[23] Zhou Y . The relationship between strategic approaches and academic procrastination in English learning among Chinese college students: the mediating effect of neuroticism. Front Educ. (2025) 10:1670701. doi: 10.3389/feduc.2025.1670701

[24] Chen W-L Chung S-H . Academic procrastination and emotion regulation: parallel trajectories and reciprocal influences over an academic semester. Pers Individ Dif. (2025) 237:113050. doi: 10.1016/j.paid.2025.11305038826717

[25] Li H Gao R Xu H . Impact of academic stress on achievement among medical postgraduates: the moderating role of the Big Five personality traits. BMC Psychol. (2026) 14:555. doi: 10.1186/s40359-026-04294-y 41814446 PMC13094071

[26] Cohen S Wills TA . Stress, social support, and the buffering hypothesis. psychol Bull. (1985) 98:310–57. doi: 10.1037/0033-2909.98.2.310 3901065

[27] Hu D Park HJ Ruberton PM Smyth JM Cohen GL . Trust in advisor predicts Ph.D. students’ academic motivation, well-being, and achievement: a prospective longitudinal study. PNAS Nexus. (2026) 5:pgaf411. doi: 10.1093/pnasnexus/pgaf411 41608137 PMC12836308

[28] Creswell JW Plano Clark VL . Designing and Conducting Mixed Methods Research. 3rd Edn. Thousand Oaks, CA: Sage Publications (2017).

[29] Ivankova NV Creswell JW Stick SL . Using mixed-methods sequential explanatory design: from theory to practice. Field Methods. (2006) 18:3–20. doi: 10.1177/1525822X05282260

[30] Van der Linden N Devos C Boudrenghien G Frenay M Azzi A Klein O . Gaining insight into doctoral persistence: development and validation of doctorate-related need support and need satisfaction short scales. Learn Individ Dif. (2018) 65:100–11. doi: 10.1016/j.lindif.2018.03.00838826717

[31] John OP Srivastava S . The Big-Five trait taxonomy: history, measurement, and theoretical perspectives. In: Pervin LA John OP , editors. Handbook of Personality: Theory and Research, 2nd Edn. Guilford Press, New York, NY (1999). p. 102–38.

[32] Wang B Andrews W Bechtoldt MN Rohrmann S de Vries RE . Validation of the short clance impostor phenomenon scale (CIPS-10). Eur J psychol Assess. (2024) 40:158–68. doi: 10.1027/1015-5759/a00074740893473

[33] Clance PR . The Impostor Phenomenon: When Success Makes You Feel Like a Fake. Toronto: Bantam Books (1985).

[34] Bobe J Wenker T Scheunemann A Fries S Bäulke L Thies DO . Delaying academic tasks and feeling bad about it: development and validation of a six-item scale measuring academic procrastination. Eur J psychol Assess. (2024) 40:59–72. doi: 10.1027/1015-5759/a00072840893473

[35] Preacher KJ Hayes AF . SPSS and SAS procedures for estimating indirect effects in simple mediation models. Behav Res Methods Instruments Comput. (2004) 36:717–31. doi: 10.3758/BF03206553 15641418

[36] Preacher KJ Hayes AF . Asymptotic and resampling strategies for assessing and comparing indirect effects in multiple mediator models. Behav Res Methods. (2008) 40:879–91. doi: 10.3758/BRM.40.3.879 18697684

[37] Hair JF Hult GTM Ringle CM Sarstedt M . A Primer on Partial Least Squares Structural Equation Modeling (Pls-Sem). 3rd Edn. Thousand Oaks, CA: Sage (2022).

[38] Braun V Clarke V . Using thematic analysis in psychology. Qual Res Psychol. (2006) 3:77–101. doi: 10.1191/1478088706qp063oa

[39] Avolio BJ Yammarino FJ Bass BM . Identifying common methods variance with data collected from a single source: an unresolved sticky issue. J Manage. (1991) 17:571–87. doi: 10.1177/014920639101700303

[40] Faul F Erdfelder E Buchner A Lang A-G . Statistical power analyses using G*Power 3.1: tests for correlation and regression analyses. Behav Res Methods. (2009) 41:1149–60. doi: 10.3758/BRM.41.4.1149 19897823

[41] Anderson JC Gerbing DW . Structural equation modeling in practice: a review and recommended two-step approach. psychol Bull. (1988) 103:411–23. doi: 10.1037/0033-2909.103.3.41127371692

[42] Henseler J Ringle C Sarstedt M . A new criterion for assessing discriminant validity in variance-based structural equation modeling. J Acad Marketing Sci. (2015) 43:115–35. doi: 10.1007/s11747-014-0403-830311153

[43] Franke G Sarstedt M . Heuristics versus statistics in discriminant validity testing: a comparison of four procedures. Internet Res. (2019) 29:430–47. doi: 10.1108/IntR-12-2017-051535579975

[44] Cain MK Zhang Z Yuan K-H . Univariate and multivariate skewness and kurtosis for measuring nonnormality: prevalence, influence and estimation. Behav Res Methods. (2017) 49:1716–35. doi: 10.3758/s13428-016-0814-1 27752968

[45] Becker J-M Cheah J-H Gholamzade R Ringle CM Sarstedt M . PLS-SEM’s most wanted guidance. Int J Contemp Hospitality Manage. (2023) 35:321–46. doi: 10.1108/IJCHM-04-2022-047435579975

[46] Hahn ED Ang SH . From the editors: new directions in the reporting of statistical results in the Journal of World Business. J World Business. (2017) 52:125–6. doi: 10.1016/j.jwb.2016.12.00338826717

[47] Rozental A Carlbring P . Understanding and treating procrastination: a review of a common self-regulatory failure. Psychology. (2014) 5:1488–502. doi: 10.4236/psych.2014.513160

[48] Steel P Klingsieck KB . Academic procrastination: psychological antecedents revisited. Aust Psychol. (2016) 51:36–46. doi: 10.1111/ap.1217340046247

[49] Huet I Casanova D . Exploring the professional development of doctoral supervisors through workplace learning: a literature review. Higher Educ Res Dev. (2022) 41:774–88. doi: 10.1080/07294360.2021.187762937339054

[50] Bai F Zhang F Xue Y . Mechanisms of anxiety among doctoral students in China. Behav Sci. (2025) 15:105. doi: 10.3390/bs15020105 40001736 PMC11851512

[51] Goundar PR Azam N Bogitini L Narayan R Devi KN . A qualitative study of doctoral students’ experiences with written supervisory feedback. Policy Futures Educ. (2026) 24:420–35. doi: 10.1177/14782103251381547

[52] Li J Xue E He Y Wang X Wu R . Obeying, following, imitating, and hoping to shape the academic habitus: supervisory interactions and doctoral students’ academic cultural (re)production. Humanities Soc Sci Commun. (2025) 12:1432. doi: 10.1057/s41599-025-05735-6

[53] Chen X Huang J . Accelerated life in academic capitalism: PhD student’s time experience in project work. Higher Educ. (2025) 90:1347–63. doi: 10.1007/s10734-024-01380-130311153

[54] Zhi H . Tangled beneath decorum: psychological contracts between master’s students and advisors in Chinese academia. Int J Educ Res Open. (2025) 9:100527. doi: 10.1016/j.ijedro.2025.10052738826717

