# Correction: When supervisor support is not enough: the roles of impostor syndrome and neuroticism in academic procrastination among doctoral students in China

**DOI:** 10.3389/fpsyt.2026.1984932

**Published:** 2026-09-18

**Authors:** Xiaohan Yang, Chong Zhang, Min Xu, Kee Jiar Yeo, Yu Liu

**Affiliations:** 1 Department of Psychology and Special Education, China National Academy of Educational Sciences, Beijing, China; 2 Department of Educational Studies and Behavioral Sciences, Faculty of Educational Sciences and Technology, Universiti Teknologi Malaysia, Johor Bahru, Malaysia; 3 School of Humanities, Southwest Jiaotong University, Chengdu, China

**Keywords:** academic procrastination, academic stress, doctoral education, higher education, impostor syndrome, mixed-methods, supervisor support

Authors “Xiaohan Yang and Chong Zhang” were erroneously omitted as equal contributing authors. These authors have contributed equally to this work and share first authorship. The original version of this article has been updated.

